# Resolution of enuresis with aripiprazole in children with psychiatric disorders: two case reports

**DOI:** 10.1186/s13256-021-02691-y

**Published:** 2021-04-21

**Authors:** Rosaria Nardello, Fulvio Guccione, Claudia Gliubizzi, Antonio Marino, Mariarita Capizzi, Salvatore Mangano

**Affiliations:** Department of Health Promotion, Mother and Child Care, Internal Medicine and Medical Specialities “G. D’Alessandro”, University of Palermo, via A. Giordano, Palermo, Italy

**Keywords:** Aripiprazole, Atypical antipsychotic, Enuresis

## Abstract

**Background:**

Aripiprazole is a third-generation atypical antipsychotic drug that acts as a stabilizer of the dopaminergic and serotonergic system. As partial agonist of the dopamine (D2) and serotonin (5-HT1A) receptors, it appears to be effective in reducing mania in patients with bipolar disorder, tics in Tourette Syndrome, aggression in schizophrenia and autism spectrum disorder. Enuresis has been reported among its side effects. Only a few studies, with conflicting results, have investigated the relationship between aripiprazole and enuresis.

**Case presentation:**

We report the disappearance of enuresis in a Caucasian girl with intellectual disability and oppositional defiant disorder and in a Caucasian boy with intellectual disability and early-onset psychosis, both following initiation of treatment with aripiprazole.

**Conclusion:**

The aim of this study was to contribute to the literature on the use of aripripazole in subjects with enuresis. Our findings lead us to suggest that aripiprazole is less burdened with side effects, including bedwetting, than other antipsychotic drugs.

## Background

Aripiprazole is a third-generation atypical antipsychotic drug that stabilizes the dopaminergic and serotonergic systems. Moreover, it is a partial agonist of the dopamine D2 receptor (D2 receptor) and serotonin (5-HT) 1A (5-HT1A) receptor and also binds to adrenergic α1, histaminic H1 and muscarinic receptors [1]. Aripiprazole is effective in reducing mania in bipolar disorder, tics in Tourette Syndrome, aggression in schizophrenia and spectrum autism disorder. Its side effects include akathisia, dizziness, drowsiness, sedation, insomnia, somnolence, weight gain, anxiety, restlessness, drooling and headache.

Enuresis has recently been reported in children with behavioural disorders on aripiprazole treatment [2–5]. However, other studies report the resolution of enuresis after the initiation of aripiprazole treatment. To our knowledge, this latter result has been reported in only five subjects, four adults and one child [6–9]

Here we report the efficacy of aripiprazole on enuresis in a girl with intellectual disability and oppositional defiant disorder and in a boy with intellectual disability and early-onset psychosis.

## Case presentation

This study was approved by the ethics committee of “Paolo Giaccone” University Hospital, Palermo, Italy. Written informed consent for publication was obtained from the patients’ parents.

The first case involves a 5-year-old Caucasian girl who was initially admitted to our clinic for behavioural disorder. She had a history of not following rules, both within and outside the family setting, and had no understanding or sense of dangers. She was unable to focus on tasks and to maintain attention on the activity on hand, interrupting these activities (e.g. playing) and tasks before completion. She refused to stop manifesting crises of anger, crying and hetero-aggressive behaviour. These behaviours occurred at home and at school, impairing relationships with peers and parents. She had an irregular sleep pattern, with frequent awakenings and primary nocturnal enuresis (5 times/week).

At 6 years of age, the girl was diagnosed with oppositional defiant disorder and treated with risperidone 1 mg/day. Three months later, her parents noticed poor clinical benefits and some side effects: hyperphagia, weight gain (8 kg), hyperprolactinaemia (42.63 ng/ml) and increasing frequency of enuresis (7 time/week). At age 7 years, we switched medication from risperidone to aripiprazole, starting with 2.5 mg/day and increasing the dose up to 5 mg/day within 2 weeks. Four weeks later, she showed improvements in her behavioural symptoms, decrease in hyperprolactinaemia and disappearance of enuresis.

The second case involves a young Caucasian boy, aged 7 years, who had been diagnosed elsewhere with generalized epilepsy secondary to a perinatal hypoxic-ischemic event, intellectual disability and early-onset psychosis. Since then, he had been treated with risperidone 1.50 mg/day, oxcarbazepine 750 mg/day and biperidene hydrochloride 100 mg/day. Soon after starting this treatment regimen, he developed daily distal tremors in the upper limbs exacerbated by emotional stress and became nonresponsive to anticholinergic treatment. At 9 years of age, nocturnal enuresis, 4 nights/week, reappeared. At 11 years of age, since the above treatment was ineffective, the proband was admitted for clinical assessment to our department where we stopped risperidone and replaced it with aripiprazole through a slow titration from 2.5 to 15 mg/day. After 1 month of treatment with the new therapeutic regimen, self-injuries and aggression decreased and bedwetting disappeared.

Both patients do not have a family history of enuresis. Moreover, urine examinations and routine biochemistry were normal.

## Discussion

Enuresis is a common elimination disorder in childhood, often associated with psychiatric disorders. Primary enuresis is defined as bedwetting by a child who has never been dry for a period of more than 6 consecutive months. Secondary enuresis is associated with the presence of enuretic symptom and occurs after a dry (no bedwetting) period of > 6 months [10].

Enuresis is regulated by the dopaminergic, noradrenergic and serotonergic systems. As such, enuresis can be a side effect of treatment with atypical antipsychotics. Multiple factors are linked to the pathophysiological mechanisms of enuresis associated with the use of atypical antipsychotics, such as a hypodopaminergic and noradrenergic deficit state in the basal ganglia, blockage of α1 adrenergic receptors, antimuscarinic effects, 5-HT4 antagonism on the activity of the detrusor muscle, 5-HT2 and 5-HT3 antagonism determining the blockage of pudendal reflexes and induction of deep sleep due to the sedative effect of the drugs [3].

To our knowledge, aripiprazole has been reported to be correlated with the appearance of enuresis in three children and two adolescents [2–5]. In contrast, in two other case reports, aripiprazole was reported to be associated with a remission of enuresis in two adult patients with clozapine-indused enuresis and in a third adult receiving combined therapy with clozapine and risperidone [6, 9]. In addition, Saliha *et al*. reported a 9-year-old boy with intellectual disability and behaviour disorders whose nocturnal enuresis cessed after the administration of aripiprazole [7], and Kantrowitz and Uzun reported the disappearance of enuresis in a schizophrenic adult after the patient had been switched from risperidone to aripiprazole [8].

Here, we present the cases of two young patients with psychiatric disorders and enuresis who were treated with aripiprazole and whose enuresis disappeared following the initiation of aripiprazole treatment. A likely explanation is that aripiprazole reduces enuresis by increasing dopamine and serotonin expression in the pathways involved in micturition and bladder contraction [1, 11, 12].

From a detailed analysis of the limited literature on this subject, we identified five patients who achieved resolution of enuresis with the administration of aripiprazole. Notably, three of these patients, who were also on other antipsychotics, were able to control the enuresis after the administration of aripiprazole.

Given also the role of serotonin receptors in the control of micturition, it could be argued that the atypical action of aripiprazole on 5-HT1A could play a role in controlling sphincter tone and bladder contraction [12].

## Conclusion

It is possible that aripiprazole may have a greater benefit on bedwetting than other antipsychotics, possibly due to its unusual action on serotonin receptors. However, to confirm this hypothesis further studies with larger case series are needed.

## Data Availability

The datasets analysed during the current study are available from the corresponding author on reasonable request.
